# PD178 Impacts Of COVID-19 On Mental Health Services: Telepsychiatry Efficacy And Substance Use Disorder Challenges In The USA

**DOI:** 10.1017/S0266462324004033

**Published:** 2025-01-07

**Authors:** Guvenc Kockaya, Selin Okcun, Ekin Begum Ozdemir, Yaren Erkut, Mustafa Kurnaz, Birol Tibet

## Abstract

**Introduction:**

The COVID-19 pandemic significantly affected mental health, particularly among individuals with existing issues, and altered mental health services. While the direct psychiatric effects of SARS‑CoV‑2 are unclear, there is potential for the virus to cross the blood-brain barrier, raising concerns about neural invasion and inflammation. This study explored these impacts and the implications for future psychiatric disorder epidemiology.

**Methods:**

This study used a mixed-methods approach, combining a literature review on pandemic-related mental health impacts with an analysis of health databases and emergency room records from the USA. It compared pre-pandemic and pandemic data on telepsychiatry, in-person services, emergency visits, and opioid incidents, employing statistical analyses to identify key trends in healthcare utilization during the COVID-19 pandemic.

**Results:**

During the COVID-19 pandemic, rates of telepsychiatry visit completion in the USA reached 74.2 percent, which was 6.68 times higher than in-person visits and indicated that telepsychiatry was an effective alternative. The use of in-person mental health services declined by 57 percent, while telehealth services increased by 1,925 percent, with a notable rise in telehealth for people with anxiety disorders. Concurrently, general emergency room visits dropped by 52 percent. In contrast, there was a 34 percent increase in opioid overdose deaths, reaching a record of 96,779 deaths in 12 months, which highlighted ongoing healthcare challenges in treating substance use disorders. The number of emergency room visits for opioid use disorder surpassed the 2019 value by May 2020.

**Conclusions:**

The pandemic significantly shifted mental health services toward telepsychiatry, proving its effectiveness, especially for anxiety disorders. Despite reduced in-person service usage, telehealth played a vital role. However, the period saw heightened challenges in substance use disorders, marked by a significant increase in opioid overdoses and emergency visits for opioid use disorder, which underscored the need for adapted healthcare strategies.

